# Bioactive Potential of a Grape Stem Blend: A Sustainable Approach to Skin Regeneration

**DOI:** 10.3390/antiox14030338

**Published:** 2025-03-13

**Authors:** Mónica Serra, Claudia Botelho, Diana Sousa, Hugo Almeida, Ana Casas, José António Teixeira, Ana Novo Barros

**Affiliations:** 1 CEB—Centre of Biological Engineering, University of Minho, 4710-057 Braga, Portugal; quality4@mesosystem.com (M.S.); claudiabotelho@deb.uminho.pt (C.B.); id5@mesosystem.com (D.S.); jateixeira@deb.uminho.pt (J.A.T.); 2 Mesosystem Investigação & Investimentos by Spinpark, Barco, 4805-017 Guimarães, Portugal; cto@mesosystem.com (H.A.); ana@mesosystem.com (A.C.); 3 LABBELS—Associate Laboratory, 4710-057 Braga, Portugal; 4 Centre of Molecular and Environmental Biology (CBMA), Aquatic Research Network (ARNET), Associate Institute of Science and Innovation for Sustainability (IB-S), University of Minho, Campus de Gualtar, 4710-057 Braga, Portugal; 5 UCIBIO, Laboratory of Pharmaceutical Technology, Faculty of Pharmacy, University of Porto, 4051-401 Porto, Portugal; 6 Associate Laboratory i4HB Institute for Health and Bioeconomy, Faculty of Pharmacy, University of Porto, 4051-401 Porto, Portugal; 7 Centre for the Research and Technology of Argo-Environmental and Biological Sciences (CITAB), Institute for Innovation, Capacity Building and Sustainability of Agri-Food Production (Inov4Agro), University of Trás-os-Montes and Alto Douro (UTAD), Quinta de Prados, 5000-801 Vila Real, Portugal

**Keywords:** circular economy, sustainability, by-product valorization, grape stems, phenolic compounds, chemical characterization, in vitro assays, biological activities, skin regeneration, antioxidant properties

## Abstract

The European wine industry is embracing sustainability through circular economy principles, particularly by valorizing by-products, such as grape stems. Grape stems are rich in phenolic compounds with recognized health benefits. This study investigates the bioactive potential of molecules extracted from a blend of grape stems (GS blend extract). The GS blend extract was chemically characterized in terms of total phenolic content (TPC), *ortho*-diphenol content (ODC), and flavonoid content (FC), with key compounds identified via HPLC-MS. The extract’s antioxidant capacity was assessed using ABTS, FRAP, and DPPH assays, while its anti-aging and depigmenting properties were evaluated through elastase and tyrosinase inhibition assays. Additionally, in vitro assays were conducted to assess its effects on skin cells, including morphology, metabolic activity, cell cycle, and cell migration. The GS blend extract was found to be rich in proanthocyanidins and exhibited notable antioxidant and depigmenting properties. In vitro assays demonstrated that the extract had no significant impact on cellular metabolic activity or cell morphology, although a reorganization of the cell monolayer was observed. Furthermore, deviations in cell migration and cell cycle regulation suggest that the GS blend extract may aid in scar formation management. Notably, the extract arrested fibroblasts in the Sub G0-G1 phase and inhibited HaCaT cell migration, supporting its potential application in cosmetic and pharmaceutical formulations aimed at scar modulation and skin health.

## 1. Introduction

The European wine sector holds a prominent position in the global economy, with countries such as France, Italy, Spain, Germany, and Portugal identified by the International Organization of Vine and Wine (OIV) as key contributors to worldwide wine production [1,2].

The global wine industry, deeply rooted in tradition and economic significance, has increasingly gained attention for its potential to support sustainable practices, particularly within the framework of circular economy principles [3,4]. Grapes, essential for wine production, not only serve as the foundation of the winemaking process but also generate valuable by-products, such as grape stems (or stalks), seeds, and skins, which hold significant potential as bioactive resources [5,6,7,8].

In the context of the circular economy [9], which emphasizes minimizing waste and maximizing resource efficiency, the valorisation of by-products becomes crucial. Grape stems, one of the most abundant by-products of winemaking (accounting for up to 14% of total by-product volume), are often overlooked despite their high content of bioactive molecules, particularly phenolics [10].

Phenolic compounds are secondary metabolites synthesized by plants in response to various environmental challenges, acting as defence mechanisms against pathogens, parasites, UV radiation, and other biotic and abiotic stressors [10,11]. Grape stems are a rich source of these compounds, including flavanols (epicatechin, catechin, quercetin-3-*O*-rutinoside), hydroxycinnamic acids (caftaric acid, coumaric acid), hydroxybenzoic acids (gallic acid), and stilbenes (viniferin), among others [12,13,14,15,16,17]. While the phenolic profile of grape stem extracts is similar across different varieties, the proportion of each compound can vary significantly, depending on factors such as soil type, maturation stage, grape size, harvest time [18,19,20,21], and extraction method [22]. The distinct proportions of phenolics in each extract significantly impact their biological potential, particularly their antioxidant, anti-inflammatory, anti-aging, anticancer, and antimicrobial properties [11,22,23].

In terms of chemical antioxidant efficacy, phenolic compounds extracted from grape stems, particularly from Touriga Nacional [13], Sousa [14], Cabernet Moravia [17], and Mazuelo [12] varieties, exhibit notable free radical scavenging capacity. Similarly, extracts from grape stems of the Mandilaria variety have demonstrated cellular antioxidant properties, especially in endothelial and muscle cells [24,25].

Beyond grape stems antioxidant activity, *Vitis vinifera* L. grape stem extracts have shown anti-aging properties by inhibiting elastase and tyrosinase enzymes, contributing to the prevention of age spots and the loss of skin elasticity and strength [26]. The inhibition of these enzymes is particularly relevant due to the role of tyrosinase in melanin synthesis, which is associated with hyperpigmentation and age spots, as well as other pigmentation-related skin disorders. Similarly, elastase is responsible for the degradation of elastin, a key structural protein in the skin. Elastin breakdown leads to reduced mechanical strength in connective tissues, wrinkle formation, and other skin deformations [26,27,28,29]. Therefore, the inhibition of these enzymes is of paramount importance in anti-aging skincare applications.

These findings highlight the diverse and promising bioactive properties of phenolic compounds extracted from grape stems, emphasizing their potential applications in health and wellness.

While previous studies have primarily focused on evaluating isolated grape varieties, this study takes a different approach by analysing the extraction of phenolic compounds from a blend of grape stems, specifically from Tinta Roriz, Touriga Nacional, Castelão, Syrah, Arinto, and Fernão Pires. Blending extracts from multiple grape varieties may have increased benefits in cosmetic skincare formulations, as different grape varieties contain distinct bioactive compounds. Their combination can result in synergistic effects that amplify skin regeneration potential [30]. For instance, resveratrol, abundant in red grape varieties, exhibits potent antioxidant and anti-inflammatory properties [31], while flavonoids, such as quercetin and catechins, found in both red and white grapes, play crucial roles in angiogenesis and extracellular matrix pathways [32,33,34,35]. Proanthocyanidins, commonly present in grape seeds and stems, contribute to collagen crosslinking [36]. and improved skin elasticity, which can further aid in minimizing scar formation.

In addition to enhancing bioactivity, blended grape extracts may offer improved stability and bioavailability. Certain polyphenols are prone to oxidation and degradation, limiting their efficacy in topical formulations. A multi-variety blend can counteract these limitations by providing a more chemically balanced and stable composition, ensuring prolonged bioactivity when applied to the skin. Moreover, a combination of bioactive molecules targets multiple wound healing pathways, creating a comprehensive approach to skin regeneration that fosters smooth, resilient, and scar-free tissue repair.

Therefore, this study explores the potential of blended grape stem extracts as an advanced cosmetic ingredient for skin regeneration. By investigating their bioactive composition, stability, and effects on skin cell function, we aim to provide insights into how a multi-variety extract can contribute to the development of next-generation skincare products.

## 2. Materials and Methods

### 2.1. Chemicals and Reagents

#### Reagents

Gallic acid (3,4,5-trihydroxybenzoic acid), Folin–Ciocalteu reagent, and acetic acid (all extra pure, >99%) were obtained from Panreac (Panreac Química S.L.U., Barcelona, Spain). Sodium hydroxide (98%), sodium nitrite, sodium carbonate (purity > 99%), aluminum chloride (purity > 99%), and ethanol were acquired from Merck (Merck, Darmstadt, Germany). Sodium molybdate (99.5%) was purchased from Chem-Lab (Chem-Lab N.V., Zedelgem, Belgium).

Additionally, catechin (98%), TROLOX (6-hydroxy-2,5,7,8-tetramethylchroman-2-carboxylic acid, purity ≥ 98.0%), ABTS ((2,2′-azino-bis-(3-ethylbenzothiazoline-6-sulfonic) acid) diammonium salt, purity ≥ 98.0%), DPPH (2,2-diphenyl-1-picrylhydrazyl radical, 100.0%), potassium persulfate (purity ≥ 99.0%), TPTZ (2,4,6-tripyridyl-s-triazine, purity ≥ 98.0%), iron (III) chloride (purity ≥ 99.9%), tyrosinase and elastase enzymes, and all reagents used in enzymatic activity assays were sourced from Sigma-Aldrich (Steinheim, Germany), while hydrochloric acid (≈37%) was obtained from Honeywell Fluka (Berlin, Germany).

All reagents used in the cell assays were obtained from PAN-Biotech. Distilled water was generated using a Millipore water purification system (Millipore, Bedford, MA, USA).

### 2.2. Preparation of GS Blend Extract

Equal amounts of grape stems from various varieties (Tinta Roriz, Touriga Nacional, Castelão, Syrah, Arinto, and Fernão Pires) were dried in an oven (Memmert, Schwabach, Germany) at 40 °C for 72 h. After drying, the stems were ground, and the resulting samples from different varieties were combined in equal proportions to create the GS blend extract.

The extraction of phenolic compounds followed the methodology defined by Dias Costa et al. [24], with certain modifications. A 1 g sample was homogenized and stirred in 50 mL of extraction solvent (ethanol/water, 70:30) for 30 min in an orbital shaker (GFL 3005, GEMINI, Apeldoorn, The Netherlands). Following homogenization, the mixture was subjected to centrifugation (Sigma 2-16KL Refrigerated Centrifuge, Sigma Laborzentrifugen, Berlin, Germany) at 10,000× *g* for 15 min at 4 °C, a process that was repeated three times. The resulting supernatants from the repeated centrifugations were pooled and adjusted to a final volume of 50 mL in a volumetric flask using the extraction solvent.

### 2.3. Determination of Phenolic Content

The phenolic content of the GS blend extract was determined using adapted spectrophotometric methodologies designed for 96-well microplates (PrimeSurface MS-9096MZ, Frilabo, Maia, Portugal), following the protocol of Gouvinhas et al. [27]. Absorbance measurements were performed using a microplate reader (Multiskan GO Microplate Photometer, Thermo Fisher Scientific, Vantaa, Finland).

### 2.4. Determination of Total Phenolic Content

The total phenolic content (TPC) of the GS blend extract was determined by mixing 1 mL of the sample with 0.5 mL of Folin–Ciocalteu reagent. Next, 2.0 mL of Na_2_CO_3_ (7.5% *w*/*v*) and 6.5 mL of water were added to the mixture. The reaction was incubated at 70 °C for 30 min, protected from light, and then cooled under running water. Absorbance was measured at 750 nm. An analytical curve was constructed using various concentrations of gallic acid, and the total phenolic content was quantified and expressed as milligrams of gallic acid equivalent per gram of sample (mg GAE/g sample).

### 2.5. Determination of Ortho-Diphenol Content

The *ortho*-diphenol content (ODC) of the GS blend extract was assessed by adding 1 mL of Na_2_MoO_4_ (50 g/L) to 4 mL of the appropriately diluted samples. After vortexing, the mixtures were left to stand at room temperature for 15 min, protected from light. Absorbance was then measured at 370 nm. Quantification was performed using gallic acid as the standard, and the results were expressed as milligrams of gallic acid equivalent per gram of dry weight (mg GAE/g dw).

### 2.6. Determination of Flavonoid Content

The method used to determine flavonoid content (FC) involved the formation of a flavonoid–aluminum complex. In this process, 0.5 mL of the diluted sample was added to a test tube, followed by 150 µL of a 5% sodium nitrite solution. After a 5 min reaction, 150 µL of a 10% aluminum chloride solution was introduced. Following a 6 min reaction period, 1 mL of a 1 M sodium hydroxide solution was added to the well. The microplate was then stirred for 30 s, and absorbance was measured at 510 nm. To construct the calibration curve, various concentrations of catechin were used. The results were expressed as milligrams of catechin per gram of dry weight of the sample (mg CAT/g dw).

### 2.7. Identification of Phenolic Compounds by HPLC-MS

The quantitative (poly)phenolic profile of the grape stem extract was determined using the methodology described by Costa-Pérez et al. [24]. Chromatographic separation of (poly)phenols was performed on a Luna C18 column (150.0 × 4.6 mm, 5.0 µm particle size, Phenomenex, Macclesfield, UK) using an Agilent HPLC 1100 series system (Agilent Technologies, Waldbronn, Germany) consisting of a binary pump (model G1312A), an autosampler (model G1313A), a degasser (model G1322A), a photodiode array (PDA) detector (model G1315B), and an ion trap spectrometer (model G2445A) with an electrospray ionization interface. The system was controlled using LCMSD software, v. 4.1 (Agilent Technologies), following the specifications detailed by Barros et al. [15].

The mobile phases used were H_2_O/formic acid (99.0:1.0, *v*/*v*) (solvent A) and acetonitrile/formic acid (9.0:1.0, *v*/*v*) (solvent B). Spectral data from all peaks were detected in the 200–600 nm range, with chromatograms recorded at 280 nm for proanthocyanidins, 330 nm for phenolic acids and stilbenes, and 360 nm for flavonols. Mass spectrometry data were acquired in negative mode. Phenolic compound identification was carried out by examining retention time (min), parent ions, and MS^2^ fragmentation patterns.

Phenolic compounds were quantified using PDA chromatograms recorded at 280 nm for proanthocyanidins, 330 nm for phenolic acids, and 360 nm for flavonols. Daily prepared calibration curves were applied using catechin (for proanthocyanidins and catechin derivatives), chlorogenic acid (for phenolic acids), and quercetin glucoside (for flavonols). The results were expressed as µg/g dw.

### 2.8. Determination of Antioxidant Capacity

The antioxidant capacity of the GS blend extract was assessed using spectrophotometric methods, specifically the ABTS ((2,2′-azino-bis-(3-ethylbenzothiazoline-6-sulfonic) acid) diammonium salt), DPPH (2,2-diphenyl-1-picrylhydrazyl), and FRAP (Ferric-Reducing Antioxidant Power) assays, according to Santos et al. [28]. All methods are based on the principle of antioxidants in the sample donating electrons to reduce the oxidized forms of the reagents.

In the ABTS and DPPH assays, the reaction involves a radical formed by the oxidation of ABTS and DPPH, respectively. In contrast, the FRAP assay is based on the reduction of a ferric complex.

#### 2.8.1. ABTS Assay

The first step of this assay involved oxidizing the ABTS salt by adding 88 µL of a 148 mM potassium persulfate solution to 5 mL of a 7 mM ABTS solution. The mixture was left to stand for 12 to 16 h at room temperature and was protected from light to achieve its most stable oxidative state.

Subsequently, the ABTS working solution was prepared by diluting the cationic ABTS solution in a 20 mM sodium acetate buffer (pH 4.5) until an absorbance of 0.700 ± 0.020 at 734 nm was obtained.

The antioxidant capacity of the samples was evaluated by adding 188 µL of the ABTS working solution to each microplate well, followed by the addition of 12 µL of the diluted sample and blank. After a 30 min of incubation at room temperature, protected from light, absorbance was measured at 734 nm [Abs (734 nm)].

For this assay, various concentrations of TROLOX were used to construct the analytical curve, with water serving as the blank. The ABTS assay measured the ability of the samples to scavenge ABTS radicals, and the inhibition percentage was calculated using the following formula:

$${\%\;\mathrm{inhibition}}_{\mathrm{Sample}}=\frac{{\mathrm{Abs}\;(734\;\mathrm{nm})}_{\mathrm{Blank}}\;-\;{\mathrm{Abs}\;(734\;\mathrm{nm})}_{\mathrm{Sample}}}{{\mathrm{Abs}\;(734\;\mathrm{nm})}_{\mathrm{Blank}}}\;\times \;100$$

The results were presented as TROLOX Equivalent Antioxidant Capacity (TEAC) and expressed in millimoles of TROLOX per gram of dry weight of the sample (mmol TROLOX/g dw).

#### 2.8.2. FRAP Assay

The evaluation of antioxidant activity using the FRAP method began with the preparation of the FRAP working solution. This solution was prepared by combining 10 volumes of 220 mM acetate buffer (pH 3.6), 1 volume of 40 mM TPTZ (2,4,6-Tripyridyl-s-Triazine) solution dissolved in 40 mM HCl, and 1 volume of 20 mM ferric chloride. The FRAP working solution, prepared daily, was preheated to 37 °C for 10 min before use.

Subsequently, 20 µL of the sample was introduced into the microplate well, followed by the addition of 280 µL of the FRAP working solution. The microplate was then shaken and incubated at 37 °C in darkness for 30 min, after which the absorbance was measured at 593 nm. To establish the calibration curve, varying concentrations of TROLOX were used. The results were expressed in millimoles of TROLOX per gram of dry weight of the sample (mmol TROLOX/g dw).

#### 2.8.3. DPPH Assay

In the initial phase of this assay, the DPPH stock solution (8.87 mM) was diluted in ethanol/water (70:30, *v*/*v*) until an absorbance close to 1.000 at 520 nm was achieved, creating the DPPH working solution.

To evaluate the antioxidant capacity of the samples, 190 µL of the DPPH working solution was added to each microplate well, followed by 10 µL of the diluted sample and blank (ethanol/water, 70:30, *v*/*v*). The mixture was incubated at room temperature for 30 min and was protected from light, and the absorbance was measured at 520 nm [Abs (520 nm)].

TROLOX, at various concentrations, was used as the standard to construct the analytical curve. The inhibition percentage was calculated using the following formula:

$${\%\;\mathrm{inhibition}}_{\mathrm{Sample}}=\frac{{\mathrm{Abs}\;(520\;\mathrm{nm})}_{\mathrm{Blank}}\;-\;{\mathrm{Abs}\;(520\;\mathrm{nm})}_{\mathrm{Sample}}}{{\mathrm{Abs}\;(520\;\mathrm{nm})}_{\mathrm{Blank}}}\;\times \;100$$

The results were presented as TROLOX Equivalent Antioxidant Capacity (TEAC) and expressed in millimoles of TROLOX per gram of dry weight of the sample (mmol TROLOX/g dw).

### 2.9. Evaluation of Anti-Aging and Depigmenting Activities

The evaluation of anti-aging activity was conducted through assays measuring the inhibition percentage of elastase and tyrosinase enzymes, following the methodology described by Taghouti et al. [29].

#### 2.9.1. Tyrosinase Inhibition Assay

The tyrosinase inhibition assay was performed to determine the percentage of tyrosinase inhibition. In this procedure, 20 μL of 1000 U/mL tyrosinase and 170 μL of a mixture containing 1 mM L-tyrosine solution, 50 mM phosphate buffer (pH 6.5), and water (in a 10:10:9 proportion) were added to the sample.

Subsequently, the microplate was incubated at 37 °C for 10 min, and the absorbance was measured at 490 nm. Positive and negative controls—1 mg/mL Kojic acid and 10% DMSO, respectively—were used.

The percentage of inhibition was calculated using the following equation:

$${\%\;\mathrm{inhibition}}_{\mathrm{Sample}}=\frac{{\mathrm{Abs}\;(490\;\mathrm{nm})}_{\mathrm{Negative}\;\mathrm{Control}}\;-\;{\mathrm{Abs}\;(490\;\mathrm{nm})}_{\mathrm{Sample}}}{{\mathrm{Abs}\;(490\;\mathrm{nm})}_{\mathrm{Negative}\;\mathrm{Control}}}\;\times \;100$$

#### 2.9.2. Elastase Inhibition Assay

The elastase inhibition assay was conducted to determine the percentage of elastase inhibition.

A 50 μL aliquot of the sample was combined with 160 μL of 0.20 mM Tris-HCl buffer (pH 8) and 20 μL of 0.80 mM N-Succinyl-Ala-Ala-Ala-p-nitroanilide substrate (prepared in Tris-HCl buffer). The mixture was incubated at room temperature for 10 min.

Subsequently, 20 μL of 1 U/L elastase enzyme (in Tris-HCl buffer) was added, and the microplate was incubated for a further 20 min at room temperature. The absorbance was then measured at 410 nm. Tris-HCl buffer was used as the negative control.

The percentage of inhibition was calculated using the following equation:

$${\%\;\mathrm{inhibition}}_{\mathrm{Sample}}=\frac{{\mathrm{Abs}\;(410\;\mathrm{nm})}_{\mathrm{Negative}\;\mathrm{Control}}-\;{\mathrm{Abs}\;(410\;\mathrm{nm})}_{\mathrm{Sample}}}{{\mathrm{Abs}\;(410\;\mathrm{nm})}_{\mathrm{Negative}\;\mathrm{Control}}}\;\times \;100$$

### 2.10. Cell Maintenance and Assays

#### 2.10.1. Cell Morphology Assay

HaCaT and BJ-5ta cells were first plated in a 48-well plate at an optimized density of 3.5 × 10^4^ cells/mL and incubated for 24 h.

Following this incubation period, the extracts were added to the wells, and incubation continued for another 24 h. The medium was then removed, and the cells were washed once with PBS. To fix the cells, 100 µL of 4% paraformaldehyde was added, followed by a 40 min incubation at room temperature (RT). The cells were then washed again with PBS.

For permeabilization, 100 µL of 0.1% Triton X-100 in PBS was added, and the cells were incubated for 30 min at RT. The permeabilization solution was prepared by mixing 5 µL of Triton X-100 with 4995 µL of PBS. After permeabilization, the cells were washed again with PBS.

Phalloidin staining was performed by diluting 0.25 µL of Alexa Fluor 568 Phalloidin (Invitrogen, A12380, Waltham, MA, USA) from a 400x stock solution into 100 µL of PBS. The staining solution was prepared by combining 3.8 µL of the Phalloidin stock solution, 1496.2 µL of PBS, and 15 mg of BSA. The cells were then incubated with this Phalloidin solution for 60 min at RT and were protected from light.

After staining, the cells were washed twice with PBS. Subsequently, 50 µL of DAPI solution was added, and the cells were incubated for 15 min. Finally, the cells were washed twice with PBS and examined using a Leica Inverted Microscope (DMI 3000B, Wetzlar, Germany, 2012).

#### 2.10.2. Cell Metabolism Assay

Cells were monitored until they reached 80% confluency. Once confluency was achieved, the DMEM medium was removed, and the cells were washed with filtered PBS (1 mL for T25 flasks). To detach the cells, 500 µL of trypsin was added to the T25 flasks, followed by incubation for 5–10 min. Detachment was confirmed by microscopy or gentle shaking. After detachment, double the volume of the medium was added to neutralize the trypsin.

For further culture, 1000 µL of the cell suspension was added to 5 mL of medium in a T25 flask. The flasks were then incubated under standard conditions.

#### Resazurin Reduction Assay as a Measurement of Cellular Metabolic Activity/Viability

In this work, the resazurin reduction assay, which is an established and widely used alternative for evaluating cell viability through metabolic activity was used. Resazurin is a non-toxic, cell-permeable compound that is reduced to the highly fluorescent resorufin by metabolically active cells. This reduction process is dependent on cellular respiration and mitochondrial function, making it a reliable indicator of cell viability.

After subculturing, the cell suspension was transferred to 15 mL Falcon tubes. A 10 µL sample was used for cell counting with a Neubauer Chamber. The average cell count from four quadrants was used to adjust the cell concentration to 4.0 × 10^4^ cells/mL.

For the assay, 200 µL of the cell suspension was plated in triplicate in a 96-well microplate and incubated for 24 h. Following incubation, the medium was removed, and 200 µL of extract at various concentrations (20.84; 41.49; 83.38; 166.75; 333.50; 667.00 μg/mL) was added, alongside controls (medium alone and medium with 0.5% DMSO). Cells were incubated for another 24 h.

The next day, the cells were washed with 1× PBS and incubated with a resazurin solution (10 mL resazurin in 1× PBS + 40 mL medium) for 2 h in the dark. Resazurin reduction was assessed by measuring the fluorescence of its reduced product, resorufin, with an excitation wavelength of 560 nm and an emission wavelength of 590 nm. The obtained results were then used to evaluate cellular viability.

#### 2.10.3. In Vitro Cell Migration Assay

The cell lines were seeded into a 24-well plate at a density of 8.0 × 10^4^ cells/mL and incubated at 37 °C in a 5% CO_2_ atmosphere until confluence was reached. A scratch was introduced into the confluent cell layer using a 10 µL pipette tip. The culture medium was then replaced with the different extract dissolved in the culture medium at a concentration of 0.5 mg/mL.

Cell migration was monitored microscopically, and images were acquired at 0 h, 6 h, and 24 h for HaCaT cells and at 0 h, 24 h, and 48 h for BJ-5ta cells. The scratch area was measured at different time points using ImageJ’s MRI Wound Healing Tool plugin (updated version 2020) [36,37].

#### 2.10.4. Evaluation of Cell Cycle by Flow Cytometry

For cell cycle analysis, cells were cultured until they reached over 90% confluency. The medium was then removed, and cells were washed with PBS. Cells were detached with 1 mL trypsin, incubated for 10 min at 37 °C in a 5% CO_2_ atmosphere, and neutralized with 2 mL of DMEM medium.

After cell counting, cells were plated in a 6-well plate in triplicate at the following densities: HaCaT: 2.64 × 10⁵ cells/well and BJ-5ta: 1.40 × 10^5^ cells/well.

Cells were incubated for 24 h to allow adherence, followed by treatment with GS blend extract for an additional 24 h. The concentrations tested were 20.84, 83.38, and 166.75 μg/mL.

Post-treatment, the medium was removed, and cells were washed with PBS. Cells were detached with 500 µL trypsin, incubated for 10 min, and neutralized with 1 mL of medium. Cells from three wells per condition were transferred into 15 mL Falcon tubes.

After cell counting, cells were centrifuged at 400× *g* for 7 min. The cell pellet was resuspended in 2 mL of an ice-cold Binding Buffer and centrifuged again at 400× *g* for 7 min. Cells were then fixed by adding 2 mL of ice-cold 70% ethanol dropwise while vortexing, followed by incubation on ice for 40 min.

Following fixation, cells were centrifuged at 400× *g* for 7 min and resuspended in a Binding Buffer, followed by another centrifugation step. The final cell pellet was resuspended in 500 µL of a staining solution (100 µL Enzyme A, 400 µL Nuclear Dye, and 10 mL Binding Buffer) and incubated in the dark for 30 min. Stained cells were analyzed for cell cycle distribution using flow cytometry.

### 2.11. Statistical Analysis

The data were analyzed using IBM SPSS 29.0 statistical software. Analysis of variance (ANOVA) was performed, followed by Tukey’s multiple range test, with significance set at *p* < 0.05.

Pearson correlation analysis was conducted using Microsoft Excel (version 16.87).

## 3. Results and Discussion

### 3.1. Phenolic Content

The phenolic (TPC), *ortho*-diphenol (ODC), and flavonoid (FC) contents of the extract obtained from the blend of grape stem varieties (GS blend extract) are presented in Table 1.

Consistent with the existing literature [24,26,38], the GS blend extract exhibits a high concentration of phenolic compounds.

It is important to take into consideration that the proportions of each grape variety present in the blend are equal, making it possible to infer the contribution of each variety to the final effect.

Leal et al. [13] characterized methanolic extracts from distinct grape stem varieties, namely, Tinta Roriz, Touriga Nacional, Castelão, Syrah, Arinto, and Fernão Pires based on their TPC, ODC, and FC contents. The authors reported that each variety exhibited distinct concentrations, ranging from 30.91 ± 0.73 to 96.12 ± 8.14 mg GAE/g dw for TPC, 32.17 ± 1.04 to 77.26 ± 5.31 mg GAE/g dw for ODC, and 25.76 ± 1.14 to 65.14 ± 0.65 mg CAT/g dw for FC.

Similarly, Silva et al. [16] determined TPC in ethanolic extracts of grape stems from Touriga Nacional and Preto Martinho, reporting values between 45.9 ± 10.7 μg/mg and 226.8 ± 6.9 μg/mg. As shown in Table 1, the chemical characterization of the GS blend extract falls within the range of methanolic extracts. However, compared to ethanolic extracts, the GS blend extract contains approximately 1.7 times higher TPC than Touriga Nacional, but it is 2.8 times lower than Preto Martinho.

The combination of grape stems can induce chemical reactions that lead to the formation of new compounds, potentially resulting in synergistic or antagonistic effects [39]. El-Belaga et al. [40] described an increase in total phenolic and flavonoid contents when grape seed and green tea extracts were mixed in equal proportions. TPC increased by approximately 4% (from 22% to 26%), while FC increased by 7% (from 53% to 60%). Conversely, Giovanelli-Vicuña et al. [34] demonstrated that mixing methanolic extracts from different fruits (grape, lemon, and blueberry) led to a significant decrease of approximately 87% in TPC and 63% in FC, highlighting the unpredictability of compound composition in a blend.

### 3.2. Identification and Quantification of Phenolic Compounds by HPLC-MS Given the Biological Relevance of Phenolic Compounds, the Detailed Phenolic Composition of the GS Blend Extract Was Analyzed Using HPLC-MS (Table 2)

As shown in Table 2, the GS blend extract is rich in flavonoids, containing 1270.47 ± 2.88 µg/g dw of flavan-3-ols, including proanthocyanidins and catechin derivatives, and 108.36 ± 0.49 µg/g dw of flavonols. These results align with the flavonoid content assay, which yielded 66.77 ± 1.96 mg CAT/g dw, demonstrating consistency between individual flavonoid quantifications and the total flavonoid content.

**Table 2:** Compounds identified in the GS blend extract and their retention times (Rt) by HPLC-MS.

Identified Compounds	Rt (min)	m/z [M-H]^−^	Quantification (μg/g dw)
**Proanthocyanidins and Catechin Derivatives**			
Proanthocyanidin trimer (B-type) (Isomer 1)	3.75	865	605.13 ± 1.48 ^a^
Proanthocyanidin trimer (B-type) (Isomer 2)	4.11	865	131.55 ± 10.68 ^c^
Proanthocyanidin trimer (B-type) (Isomer 3)	6.16	865	229.67 ± 1.7 ^b^
Proanthocyanidin trimer (B-type) (Isomer 4)	6.33	865	48.48 ± 1.27 ^e^
Proanthocyanidin trimer monogallate (Isomer 1)	7.11	1017	145.83 ± 3.56 ^c^
Proanthocyanidin trimer monogallate (Isomer 2)	9.95	1017	67.43 ± 0.27 ^d^
Epicatechin-3-*O*-glucoside	11.35	449	42.38 ± 1.19 ^e^
**Total**			1270.47 ± 2.88
**Flavonols**			
Rutin	10.10	609	9.82 ± 0.08 ^c^
Quercetin-3-*O*-glucoside (Isomer 1)	10.42	463	20.16 ± 0.29 ^b^
Isorhamnetin-3-*O*-glucoside	10.64	301	66.38 ± 0.22 ^a^
Kampferol-3-*O*-glucoside	11.42	447	12.01 ± 1.36 ^c^
**Total**			108.36 ± 0.49
**Stilbenes**			
Resveratrol tetramer (Isomer 1)	14.75	905	14.41 ± 0.39
**Total**			14.41 ± 0.39

Rt, retention time; dw, dry weight, LOQ, limit of quantification Distinct letters in the same row indicate significant differences between varieties (*p* < 0.05), according to the Welch test followed by a post-hoc Games-Howell test.

The *ortho*-diphenol content was confirmed through the identification of catechin and proanthocyanidins. The predominant compounds in the GS blend extract include proanthocyanidin trimers (B-type) (Isomers 1, 2, and 3), proanthocyanidin trimer monogallates (Isomers 1 and 2), and various catechin derivatives.

Leal et al. [13] identified flavan-3-ols (catechin, epicatechin), flavonols (rutin), and stilbenes (resveratrol, viniferin) in methanolic extracts of grape stems from Tinta Roriz, Touriga Nacional, Castelão, Syrah, Arinto, and Fernão Pires. Similarly, Silva et al. [16] identified flavan-3-ols (catechin, epicatechin, gallo-catechin gallate, catechin gallate), flavonols (rutin, quercetin 3-*O*-galactoside, quercetin 3-*O*-glucoside, quercetin 3-*O*-rhamnoside), and stilbenes (trans-resveratrol) in ethanolic extracts of grape stems from Touriga Nacional and Preto Martinho.

In the GS blend extract, the formation of derivatives is particularly notable, with the identification of proanthocyanidin trimers and resveratrol tetramer. This suggests that chemical interactions during the blending process resulted in the formation of complex compounds, which may explain the absence of catechin, epicatechin, and resveratrol while their derivatives are present. This reinforces the assumption that these compounds underwent complexation, forming trimers and tetramers.

Additionally, Leal et al. [13] identified phenolic acids in different grape stem extracts, including gallic acid (Syrah, Arinto, Fernão Pires), protocatechuic acid (Touriga Nacional, Castelão, Arinto, Fernão Pires), and cinnamic and caftaric acids (all varieties). Similarly, Silva et al. [16] identified gallic acid, coumaric acid, vanillic acid, chlorogenic acid, protocatechuic acid, and ferulic acid in grape stem extracts from Touriga Nacional and Preto Martinho. However, no phenolic acids were detected in the GS blend extract. This discrepancy may be due to chemical interactions or transformations occurring during the blending process, potentially leading to the alteration or degradation of these compounds, impairing their detection. El-Beltagi et al. [40] suggested that proanthocyanidins can combine with gallic acid, forming gallate esters and glycosides, as previously observed in grape seed and green tea extract mixtures. The presence of proanthocyanidin derivatives, such as proanthocyanidin trimer monogallate, supports this assumption.

### 3.3. Radical Scavenging Capacity and Reducing Power of GS Blend Ectract 

The antioxidant activity of the GS blend extract is a key indicator of its biological potential. To evaluate this, three distinct methods were employed: ABTS, DPPH, and FRAP assays. ABTS and DPPH assays assess a sample’s ability to scavenge free radicals. However, DPPH is more responsive to lipophilic compounds, while ABTS is a more versatile method, measuring both hydrophilic and lipophilic antioxidants. The FRAP assay evaluates antioxidant potential by measuring the extract’s ability to reduce ferric (Fe^3+^) ions to ferrous (Fe^2+^) ions. The results of these analyses are presented in Table 3.

The GS blend extract demonstrated strong antioxidant activity, particularly in the ABTS assay, indicating a rich presence of hydrophilic phenolic compounds capable of scavenging free radicals, including glucosides. Several hydrophilic compounds were identified in the GS blend extract, such as rutin, quercetin-3-*O*-glucoside (Isomer 1), isorhamnetin-3-*O*-glucoside, kaempferol-3-*O*-glucoside, and epicatechin-3-*O*-glucoside.

The antioxidant potential of various grape stem varieties, Tinta Roriz, Touriga Nacional, Castelão, Syrah, Arinto, and Fernão Pires, has been reported to vary across different assays: 0.35 ± 0.00 to 0.84 ± 0.06 mmol T/g in the ABTS assay, 0.15 ± 0.01 to 0.64 ± 0.05 mmol T/g in the DPPH assay, and 0.35 ± 0.02 to 1.03 ± 0.06 mmol T/g in the FRAP assay. Costa et al. [24] conducted a similar study on another set of grape stem varieties, including Arinto, Códega do Larinho, Esgana Cão, Folgasão, Fernão Pires, and Moscatel, and found comparable results using the ABTS, DPPH, and FRAP assays.

The difference in antioxidant activity between the GS blend extract and isolated grape varieties can be attributed to the complex interactions among the phenolic compounds present in each grape variety. Phenolic compounds, such as proanthocyanidins and resveratrol, can form complex structures that either enhance or diminish antioxidant activity. Kotov et al. [41] observed a synergistic effect when combining plant extracts, such as bur-marigold and hawthorn, leading to a 110.6% increase in antioxidant activity. Similarly, another three-component mixture, bur-marigold, calendula, and hawthorn, showed a 112% increase. Aslam et al. [42] also reported enhanced antioxidant potential when combining plant extracts, particularly mixtures of *Terminalia arjuna*, *Rauvolfia serpentina*, *Elettaria cardamomum*, and *Crataegus oxyacantha*.

However, not all extract combinations result in increased antioxidant activity. Giovagnoli-Vicuña et al. [43] found an antagonistic interaction in a fruit mixture extract (grape, lemon, and blueberry). Similarly, Peixoto et al. [44] noted that grape seeds exhibited a higher concentration of phenolic compounds and greater antioxidant potential compared to pomace (a mixture of grape skins and seeds), indicating that the combination of these components can result in diminished antioxidant capability.

Plumb et al. [45] explored how polymerization, galloylation, and glycosylation affect the antioxidant activity of catechins and proanthocyanidins. Using the TBARS assay for lipid-phase antioxidant activity and the ABTS assay for aqueous-phase activity, they discovered that catechins polymerized from monomers to tetramers, decreasing the ability to prevent free radical damage in lipidic systems. A monomeric catechin presented an IC50 of 3.4 µM in the lipid peroxidation assay, while trimers had a higher IC50 of 6.5 µM, indicating reduced effectiveness.

In contrast, antioxidant activity in the aqueous phase increased with polymerization. Monomers exhibited a TROLOX Equivalent Antioxidant Capacity (TEAC) value of 2.47, whereas trimers displayed a significantly higher TEAC value of 4.87. This trend clearly demonstrates that polymerization enhances hydrophilic antioxidant activity while diminishing lipophilic protection, suggesting a structural influence on antioxidant partitioning between lipid and aqueous environments.

These findings have important implications for the bioavailability and functional application of catechin-based antioxidants. In lipid-rich environments, such as cell membranes and lipoproteins, monomeric catechins may provide more effective protection against oxidative stress. However, in aqueous systems, like blood plasma and intracellular compartments, polymerized catechins may exhibit superior antioxidant potential. This suggests that the degree of polymerization plays a crucial role in determining the efficacy of catechin-derived antioxidants in different biological contexts.

The results obtained in this study align with the findings of Plumb et al. [45]. The elevated antioxidant activity measured by ABTS, uncommon in isolated varieties, is notably higher in the GS blend extract, likely due to the presence of proanthocyanidin trimers.

These findings suggest that phenolic compounds exert their antioxidant effects through different mechanisms, depending on the environment. Therefore, antioxidant effectiveness cannot be solely explained by the ability of phenolic hydroxyl groups to donate hydrogen. Antagonistic effects in mixtures may occur due to interactions between phenolic compounds, such as the formation of hydrogen bonds. As the mixture oxidizes, it is possible to regenerate into free radicals, which may then accept electrons or hydrogen atoms from other antioxidants within the mixture, further influencing antioxidant activity.

### 3.4. Assessment of Anti-Ageing and Skin Depigmentation Effects

The anti-aging and depigmentation properties of the GS blend extract were evaluated using enzyme inhibition assays for elastase and tyrosinase.

The evaluation of anti-aging and depigmentation properties using elastase and tyrosinase inhibition assays is scientifically justified due to the critical roles these enzymes play in skin aging and pigmentation.

Elastase is an enzyme responsible for breaking down elastin, a key structural protein that maintains skin elasticity and firmness. Overactive elastase leads to the degradation of elastin fibers, contributing to visible signs of aging such as wrinkles, sagging, and loss of skin resilience [46,47]. Similarly, tyrosinase is a crucial enzyme involved in melanin synthesis, catalyzing the oxidation of tyrosine to dopaquinone, which eventually leads to pigment formation in the skin. Excessive tyrosinase activity results in hyperpigmentation conditions such as dark spots, melasma, and uneven skin tone. Compounds that inhibit tyrosinase can effectively reduce melanin production, leading to skin brightening and depigmentation [48].

The results of elastase and tyrosinase inhibition by the GS blend extract are presented in Table 4.

As seen in Table 4, the GS blend extract exhibits low elastase inhibition ability but demonstrates an average tyrosinase inhibition capability (approximately 46%). When comparing these results with the literature, the limited ability of the GS blend extract to inhibit elastase becomes evident. Leal et al. [13] reported that isolated grape varieties exhibit elastase inhibition percentages ranging from 73% to 98%. Similarly, Jiratchayamaethasakul et al. (2020) showed that *S. europaea* extract had the highest anti-elastase activity (74.88 ± 4.84%) among 22 halophyte plant extracts, further highlighting the weak elastase inhibition capability of the GS blend extract.

On the other hand, the studies conducted by Leal et al. [13] and Jiratchayamaethasakul et al. [47] support the good performance of the GS blend extract in inhibiting tyrosinase, with inhibition values ranging from 44% to 54% for isolated grape stem extracts and 58.62 ± 6.08% for *S. anglica* [47].

This difference can be attributed to the complex interactions among phenolic compounds present in the extract. Yu et al. [49] demonstrated the inhibitory capacity of isolated phenolic compounds, such as cinnamic acid, ferulic acid, quercetin, isorhamnetin, and gallic acid, both individually and in combination. The distinct combination of these phenolic compounds led to a variety of results due to chemical interactions, which may or may not exert a synergistic effect on tyrosinase inhibition. Yu et al. [49] also demonstrated that quercetin, cinnamic acid, and ferulic acid have a synergistic effect on tyrosinase inhibition.

These findings suggest that phenolic compounds can interact in ways that either enhance or diminish the function of tyrosinase. The low elastase inhibition observed in the GS blend extract may be due to less optimal combinations or interactions of phenolic compounds compared to those found in isolated grape varieties.

### 3.5. Pearson Correlation

The Pearson correlation coefficient, also known as the product moment correlation coefficient, is dimensionless and ranges from −1 to +1. A positive value indicates a positive relationship, while a negative value indicates a negative relationship. A perfect correlation, where all data points on a scatter plot lie on a straight line, is represented by a coefficient of +1 (perfect positive) or −1 (perfect negative). A coefficient of zero indicates no linear relationship between the variables, meaning they are uncorrelated [50].

To understand the correlation between the different properties assessed in this study, a Pearson correlation analysis was performed (Table 5).

As observed in Table 5, TPC is positively correlated with ODC and FC, showing a near-perfect positive correlation. This result was expected, as *ortho*-diphenols and flavonoids belong to the phenolic compound family, which directly contributes to the increase in TPC. Consequently, as TPC increases, there is a simultaneous rise in ODC and FC, demonstrating a co-dependent relationship. This interdependence arises from the structural similarities and shared biosynthetic pathways of phenolic compounds, which collectively influence antioxidant properties and enzyme inhibition potential.

The total phenolic content (TPC, ODC, and FC) also appears to be positively correlated with antioxidant activity as measured by the ABTS assay, though it is only moderately correlated with tyrosinase enzyme inhibition. This indicates that phenolic compounds play a significant role in scavenging free radicals in aqueous-phase systems, which is effectively captured by the ABTS method. The moderate correlation with tyrosinase inhibition suggests that while phenolics contribute to the extract’s depigmenting properties, other bioactive compounds or synergistic interactions may further enhance tyrosinase inhibition.

Conversely, a negative correlation is observed between phenolic content and both elastase enzyme inhibition and antioxidant activity, as measured by the DPPH assay. This suggests that while the GS blend extract effectively neutralizes radicals in hydrophilic environments (as shown by the ABTS assay), its efficacy in lipophilic antioxidant systems (as measured by DPPH) may be limited. This could be due to the hydrophilic nature of many phenolic compounds, which may have a lower affinity for lipid-phase radical scavenging. Similarly, the negative correlation with elastase inhibition suggests that phenolics alone may not be the primary contributors to elastase inhibition, implying that other classes of compounds may be responsible for anti-aging effects related to elastin preservation.

Overall, these findings demonstrate that the phenolic compounds present in the GS blend extract are primarily responsible for its antioxidant activity against free radicals in aqueous environments and its inhibitory effect on tyrosinase, supporting its potential application in skin-brightening formulations. However, the weaker correlation between elastase inhibition and lipid-phase antioxidant activity suggests that additional bioactive compounds may be necessary to enhance its anti-aging properties. Further studies focusing on the identification and characterization of these compounds, as well as their interactions with phenolics, would be valuable for optimizing the extract’s efficacy in both antioxidant and anti-aging applications.

The work of Ali et al. [51] corroborates these findings, as they demonstrated a Pearson coefficient close to 1 when correlating total phenolic content with flavonoid content in a *Caragana brachyantha* extract. Additionally, phenolic content showed a moderate correlation with tyrosinase inhibition. However, contrary to the results of this study, phenolic content appeared to be strongly correlated with antioxidant activity, as measured by the FRAP assay. Similarly, Teixeira et al. [52] reported a significant correlation between TPC and ABTS (0.912) as well as FRAP (0.944), along with a notable inverse correlation in DPPH assays (−0.848).

### 3.6. In Vitro Assays

The skin consists of multiple layers, each with distinct characteristics and specific cell populations. Among these, keratinocytes and fibroblasts were selected for this study due to their fundamental roles in skin integrity and repair. Keratinocytes, located in the epidermis, are essential for maintaining epithelial cohesion and providing protection against external factors. In contrast, fibroblasts, found in the dermis, play a key role in synthesizing structural proteins, such as collagen and elastin, which contribute to skin firmness and elasticity [53].

With aging, the epidermis becomes 10–50% thinner, primarily due to keratinocyte atrophy. Another hallmark of aging is the accumulation of senescent cells and an increase in cellular apoptosis [54,55,56]. In the dermis, aging is also characterized by a reduction in fibroblast number, size, and cytoplasmic extensions. These changes are associated with alterations in the endoplasmic reticulum, diminished proliferative and metabolic activity, and a decreased expression of extracellular matrix (ECM) components, particularly collagen and elastin [57,58,59].

In this context, several cellular assays were performed using a keratinocyte cell line (HaCaT) and a fibroblast cell line (BJ-5ta) to assess the effects of the GS blend extract on distinct aspects of cellular behaviour.

#### 3.6.1. Influence of the GS Blend Extract on Cell Morphology

**Figure 1:**
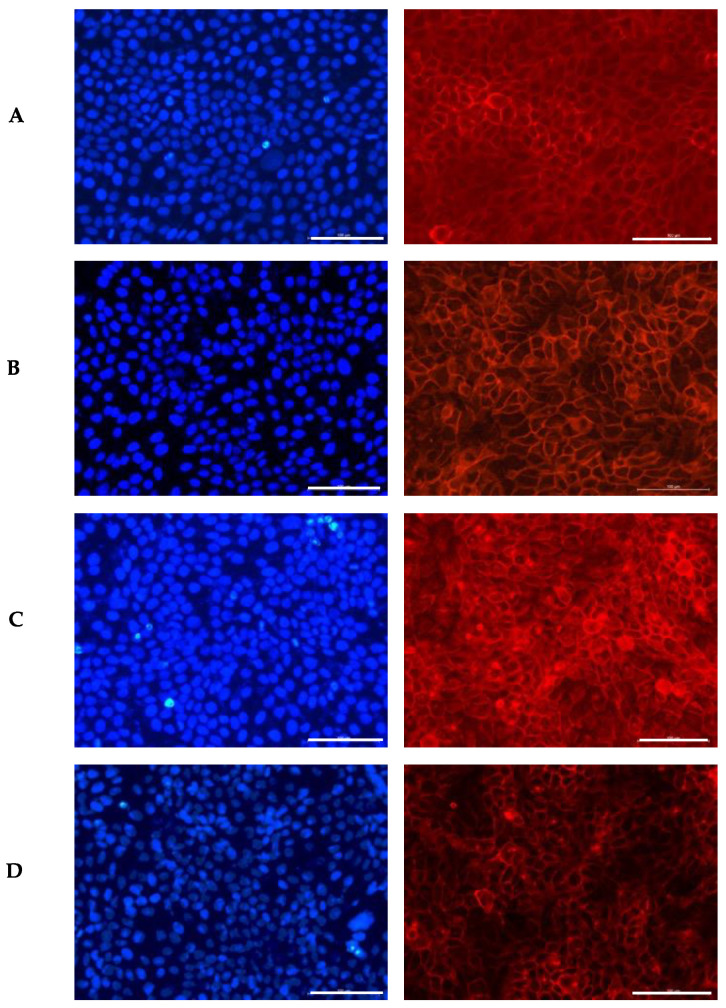
Influence of the GS blend extract on keratinocyte (HaCaT cells) morphology after 24 h of exposure. (**A**) DMSO control, showing a uniform distribution of well-spread, flattened cells with intact cytoskeletal organization, (**B**) 20.84 µg/mL GS blend extract, where slight alterations in cell shape and distribution begin to appear, (**C**) 83.38 µg/mL GS blend extract, showing increased cytoskeletal disorganization and reduced cell spreading, (**D**) 166.75 µg/mL GS blend extract, exhibiting pronounced loss of cell–cell interaction and altered spatial orientation. The actin cytoskeleton is stained in red (Alexa Fluor 568 Phalloidin), and nuclei are stained in blue (DAPI). Scale Bar 100 μm.

Keratinocytes have a polygonal shape, appearing cuboidal in the basal layer and becoming increasingly flattened as they migrate towards the surface. These cells contain a prominent nucleus that gradually diminishes in size during maturation, reflecting their transition to a keratinized state [60,61]. Given the critical role of keratinocyte morphology in maintaining skin structure, the impact of the GS blend extract on this feature was examined. The results are illustrated in Figure 1.

In the control group, the cells appeared flattened, with a uniform distribution of their prominent nuclei (blue) and cytoplasmic proteins (red). The presence of the GS blend extract appeared to induce disorganization in cell spatial orientation in a dose-dependent manner.

Similarly, George et al. [62] examined the impact of luteolin (5–80 μM) on the same keratinocyte line used in this study, HaCaT. Their study revealed that luteolin treatment led to a significant loss of cell–cell adhesion, causing the cells to lose their normal connections with each other. Additionally, they observed the development of membrane protrusions and distended areas on the cell surface, as well as the formation of fluid-filled spaces within the cells, indicative of cellular distress. Likewise, Hsu et al. [63] reported that keratinocytes treated with epigallocatechin-3-gallate (50 µM) underwent morphological changes, including an increase in enlarged, flattened, squame-like cells, which was attributed to keratinocyte differentiation. D’Angelo et al. [64] also documented morphological alterations in HaCaT cells treated with 250 µM and 500 µM of Annurca Apple polyphenol extract, including dissociation from the growth substrate, nuclear disintegration, and a decrease in cellular size. These changes were linked to the extract’s strong inhibitory effect on keratinocyte growth, highlighting its potential for treating psoriasis.

**Figure 2:**
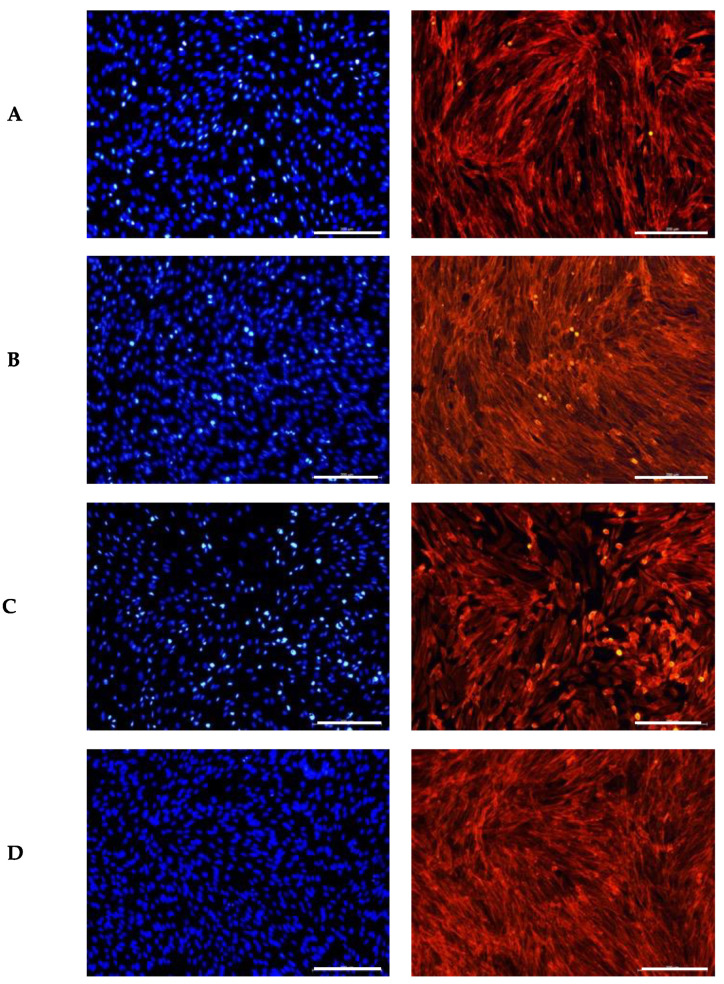
Influence of the GS blend extract on fibroblast (BJ-5ta cells) morphology after 24 h of exposure. (**A**) DMSO control, showing a uniform distribution of spindle-shaped cells with intact cytoskeletal organization, (**B**) 20.84 µg/mL GS blend extract, (**C**) 83.38 µg/mL GS blend extract, and (**D**) 166.75 µg/mL GS blend extract, exhibiting decreased cytoskeletal organization and altered spatial orientation in a dose-dependent manner. The actin cytoskeleton is stained in red (Alexa Fluor 568 Phalloidin), and nuclei are stained in blue (DAPI). Scale Bar 100 μm.

Fibroblasts are characterized by their plump, spindle-shaped, or stellate forms and centrally located oval or round nuclei [65]. A similar approach was used to evaluate the impact of the GS extract blend on a fibroblast cell line, as shown in Figure 2. The GS extract blend appeared to have a similar effect on fibroblast morphology as observed in keratinocytes.

Czemplik et al. [66] reported comparable changes in normal human dermal fibroblast (NHDF) morphology when treated with *Linum usitatissimum* extract (140.4 μg TPC). The treated cells exhibited a rounded shape instead of their typical elongated form. However, the nuclei remained similar in size and shape to those in the control group, consistent with our observations. The authors attributed these morphological alterations to the toxicity of high concentrations of phenolic compounds.

Similarly, Kikowska et al. [67] observed that human skin fibroblasts treated with a callus extract at lower concentrations (12.5 μg/mL) maintained a proper elongated shape with large, round nuclei. In contrast, fibroblasts exposed to higher concentrations (100 μg/mL) exhibited abnormal morphology, lacking the typical elongations. Both studies corroborate the results observed in this study.

In conclusion, although the GS blend extract does not appear to significantly alter cell morphology, it does induce noticeable changes in the organization of the cell monolayer.

#### 3.6.2. Influence of the GS Blend Extract on Cellular Metabolic Activity

To assess the influence of the GS blend extract on cells, the metabolic activity of keratinocytes (HaCaT) and fibroblasts (BJ-5ta) was evaluated following treatment with the extract.

**Figure 3:**
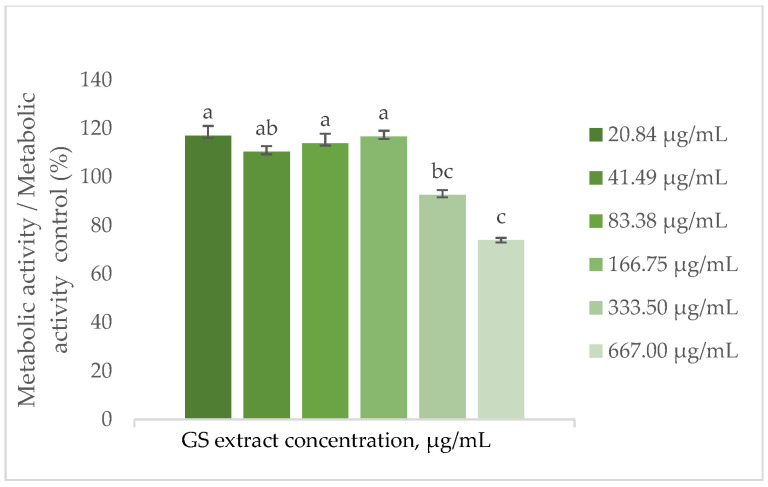
Effect of the GS blend extract on the metabolic activity of keratinocytes (HaCaT cell line) measured by the resazurin reduction assay after 24 h of exposure to different concentrations. The GS blend results are presented as mean ± standard error (n = 3). Significant differences between samples are denoted by different lowercase letters (*p* < 0.05).

The findings depicted in Figure 3 demonstrate that the metabolic activity of HaCaT cells increases by approximately 15% in the presence of the GS blend extract, with a threshold concentration of 166.75 µg/mL. Above this concentration, metabolic activity decreases, reaching a minimum of 30% at the highest concentration tested (667 µg/mL).

**Figure 4:**
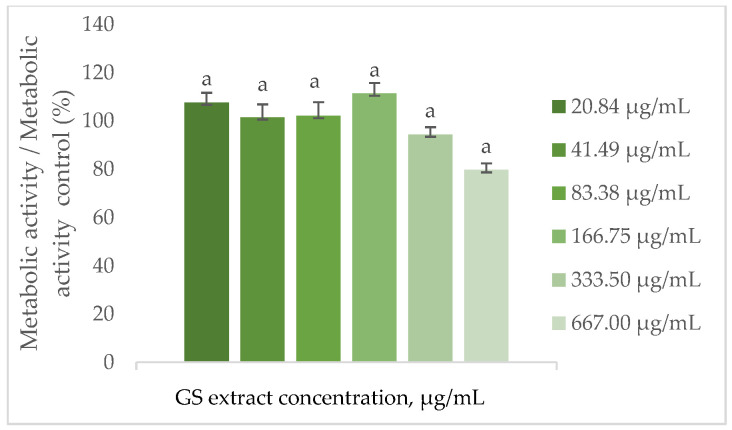
Effect of the GS blend extract on the metabolic activity of fibroblasts (BJ-5ta cell line) measured by the resazurin reduction assay after 24 h of exposure to different concentrations. The GS blend results are presented as mean ± standard error (n = 3). Significant differences between samples are denoted by a lowercase letter (*p* < 0.05).

As shown in Figure 4, the metabolic activity of BJ-5ta cells remains statistically unchanged when treated with the GS blend extract up to 667.00 µg/mL.

This observation is supported by Domínguez-Perles et al. [63], who investigated the cytotoxic effects on HaCaT keratinocytes treated with extracts from various grape stem varieties (Tinto Cão, Tinta Barroca, Moscatel Branco, Malvasia Fina) across a range of concentrations (0.04–30.60 ng GAE/mL). Their study demonstrated that the cells’ metabolic activity exceeded 97.5% compared to the control (100%).

Similarly, Sangiovanni et al. [68] analysed cell metabolic activity upon contact with leaves of the *Teinturiers* variety in HaCaT cells. Their study revealed no signs of cytotoxicity in cells exposed to the extract within a concentration range of 5 to 500 µg/mL.

The metabolic activity results suggest that the GS blend extract is compatible with the evaluated cell lines. While it does not significantly affect fibroblast metabolism, it appears to promote metabolic activity in keratinocytes at moderate concentrations but reduces it at higher concentrations.

#### 3.6.3. Influence of the GS Blend Extract on the Keratinocyte and Fibroblast (HaCaT and BJ-5ta, Respectively) Cell Cycle

The cell cycle is a fundamental process by which cells grow, replicate their DNA, and divide into two daughter cells. This cycle is essential for the growth, development, and repair of multicellular organisms. It consists of several distinct phases that ensure precise replication and segregation of genetic material, as well as proper cell growth [69].

The Sub G0-G1 phase typically indicates the presence of apoptotic or dead cells, characterized by reduced DNA content due to fragmentation. In contrast, the G0-G1 phase includes cells that are either resting or in the initial stage of the cell cycle. During the S phase, cells undergo active DNA replication, reflecting a heightened level of proliferative activity as they prepare for division. Finally, in the G2-M phase, cells complete DNA synthesis and prepare for mitosis, the final step in cell division [70,71].

Understanding these phases is crucial for evaluating cell proliferation dynamics and the cellular response to treatments. Thus, the effect of the GS blend extract on the HaCaT cell cycle was assessed using flow cytometry. Fluorescence intensity, which is proportional to DNA content, allowed for the distinction of cells in different phases of the cycle. Apoptotic cells exhibited reduced DNA content compared to viable cells in the G0/G1 phase.

**Figure 5:**
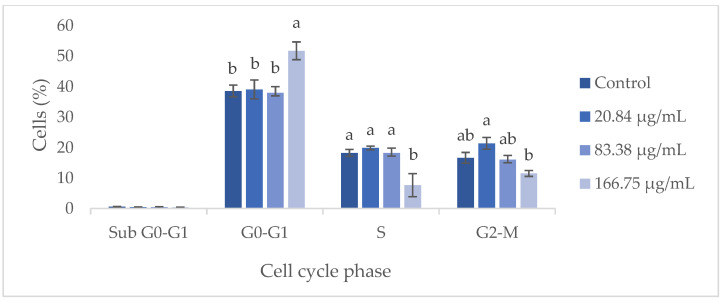
Flow cytometry analysis of the cell cycle in keratinocytes (HaCaT cell line) following exposure to different concentrations of the GS blend extract. The GS blend results are presented as mean ± standard error (n = 3). Significant differences between samples for each cell cycle phase are denoted by different lowercase letters (*p* < 0.05).

Flow cytometry analysis demonstrated that the GS blend extract does not significantly influence the HaCaT cell cycle at concentrations ranging from 20.84 to 83.38 µg/mL (Figure 5). However, at a higher concentration (166.75 µg/mL), an arrest in the G0-G1 phase was observed, leading to a reduction in the number of cells in the S phase. This suggests that at higher concentrations, the GS extract can inhibit cell proliferation by preventing cells from progressing to the S phase, thereby blocking DNA replication and subsequent cell division. Furthermore, when cells were treated with the GS extract at concentrations above 166.75 µg/mL, a 70–100% decrease in metabolic activity was observed, potentially indicating a reduction in cell number due to the inhibition of proliferation or induction of apoptosis. 

**Figure 6:**
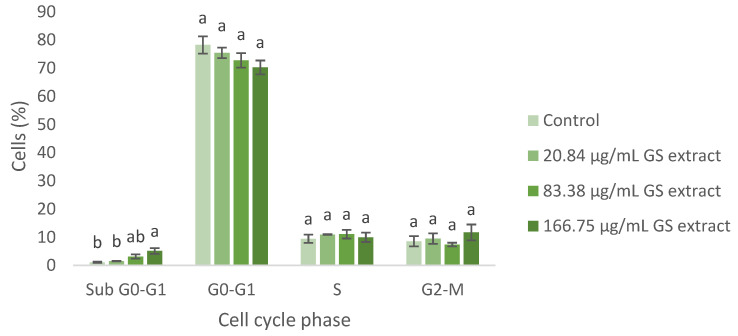
Flow cytometry analysis of the cell cycle in fibroblasts (BJ-5ta cell line) following exposure to different concentrations of the GS blend extract. The GS blend results are presented as mean ± standard error (n = 3). Significant differences between samples for each cell cycle phase are denoted by different lowercase letters (*p* < 0.05).

The cell cycle of BJ-5ta fibroblast cells was also evaluated using flow cytometry, with the fibroblast gate selected in the same manner. The results are shown below (Figure 6).

Regarding the BJ-5ta cell cycle, the GS blend extract does not appear to significantly alter cell cycle progression. However, at higher concentrations (83.38 and 166.75 µg/mL), cell cycle arrest in the Sub G0-G1 phase was observed, suggesting the potential induction of apoptosis (Figure 6).

These findings support the potential of the GS extract in managing scar formation. The GS extract arrests keratinocytes and fibroblasts in the G0-G1 and Sub G0-G1 phases, impeding proliferation and promoting apoptosis. This is crucial for managing keloids by reducing excessive fibrotic responses [71,72,73]. Similarly, treating hypertrophic scars involves inhibiting fibroblast proliferation and inducing apoptosis [70].

Overall, these findings highlight the therapeutic potential of the GS extract in managing scar formation by regulating the cell cycle and promoting apoptosis.

#### 3.6.4. Effect of the GS Blend Extract on Cell Migration of HaCaT Keratinocytes and BJ-5ta Fibroblasts

**Figure 7:**
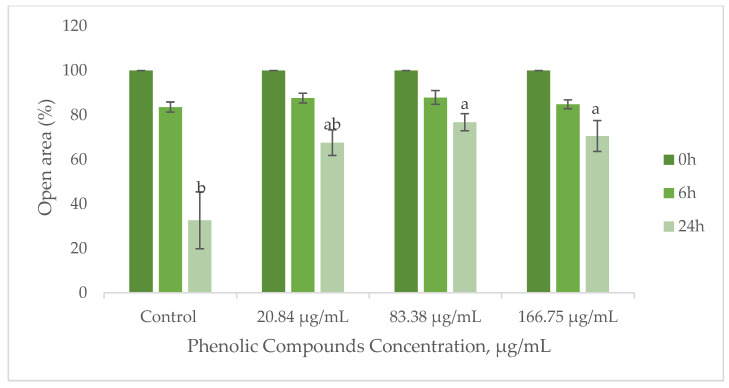
Influence of the GS blend extract on keratinocyte (HaCaT cell line) migration measured using the scratch assay at 0, 6, and 24 h. The GS blend results are presented as mean ± standard error (n = 3). Significant differences between samples at 24 h are denoted by different lowercase letters (*p* < 0.05).

Cell migration is a crucial process in wound healing, involving the movement of keratinocytes to cover the wound site and fibroblasts to deposit collagen and remodel the extracellular matrix [74,75]. To evaluate the influence of the GS extract on wound healing, the migratory ability of HaCaT keratinocytes and BJ-5ta fibroblasts was assessed using the scratch method. Figure 7 demonstrates the migration performance of HaCaT cells upon contact with the GS blend extract.

The results suggest that the GS blend extract does not affect BJ-5ta fibroblast migration but reduces HaCaT cell migration in a concentration-dependent manner (Figure 7).

At a lower concentration (20.84 µg/mL), HaCaT cell migration remained comparable to that of the control group, indicating no adverse effects. However, at higher concentrations (83.38–166.75 µg/mL), the extract significantly reduced migration, suggesting potential applications in the controlled inhibition of cell movement.

**Figure 8:**
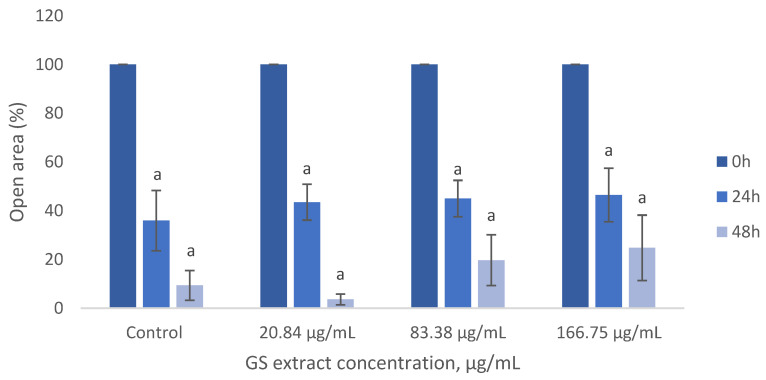
Influence of the GS blend extract on keratinocyte (BJ-5ta cell line) migration measured using the scratch assay at 0, 6, and 24 h GS blend. The results are presented as mean ± standard error (n = 3). Significant differences between samples at 24 h and samples at 48 h are denoted by a lowercase letter (*p* < 0.05).

As shown in Figure 8, the GS blend extract does not significantly impact BJ-5ta fibroblast migration, as migration rates remained similar across treated and untreated cells.

Research by Loggenberg et al. [72] demonstrated that plant-based extracts can suppress keratinocyte migration by reducing platelet-derived growth factor (PDGF-AA) expression, which plays a key role in keratinocyte proliferation and angiogenesis. Since angiogenesis is a major factor in hypertrophic scars and keloids, the inhibition of keratinocyte migration by the GS extract suggests potential anti-angiogenic properties, which could help control excessive scar formation [73,74].

Studies indicate that keloids and hypertrophic scars exhibit increased angiogenesis, suggesting that targeting angiogenic pathways may contribute to reducing excessive scar formation [75,76,77,78]. Thus, the observed reduction in HaCaT cell migration may be linked to anti-angiogenic mechanisms, supporting GS extract’s potential application in scar therapy.

The GS blend extract does not promote wound healing through increased cell migration; its ability to inhibit migration and proliferation aligns with findings related to scar tissue regulation, suggesting potential therapeutic applications in controlling keloids and hypertrophic scars.

While our study provides preliminary evidence that the GS blend extract influences key cellular behaviours involved in scar modulation, particularly through the inhibition of keratinocyte migration and proliferation, it is important to mention that scar formation is largely influenced by extracellular matrix (ECM) remodelling. Abnormal ECM deposition, including altered collagen type I and III expression, matrix metalloproteinase (MMP) activity, and tissue inhibitors of metalloproteases (TIMPs), plays a pivotal role in fibrosis and scar formation. Taking into consideration the promising results obtained in this study, further research will be conducted to investigate GS extract’s direct effects on ECM remodelling, namely, by assessing the extract’s impact on collagen synthesis, MMP/TIMP balance, and collagenase.

## 4. Conclusions

This study comprehensively analysed the GS blend extract, focusing on its phenolic content, antioxidant properties, and biological effects. The extract was found to be rich in bioactive phenolic compounds, particularly proanthocyanidin trimers, catechins, rutin, quercetin, and resveratrol tetramers. The GS extract exhibited strong antioxidant activity, particularly in the ABTS assay, likely due to its high content of proanthocyanidin trimers, which are known for their potent radical scavenging properties.

In in vitro assays, the GS extract was non-cytotoxic at low concentrations. However, at higher concentrations, it inhibited cell proliferation and migration, particularly in HaCaT keratinocytes, induced cell cycle arrest in the G0-G1 and Sub G0-G1 phases, and increased fibroblast apoptosis.

These findings suggest that the GS extract may play a role in regulating cell proliferation. However, the data do not support its direct application in wound healing or scar formation, particularly in keloids, as no evidence of extracellular matrix remodelling was provided. Further studies are needed to clarify its mechanisms of action.

## Figures and Tables

**Table 1:** Total phenolic, *ortho*-diphenol, and flavonoid content of the GS blend extract determined by colorimetric chemical assays.

TPC (mg GAE/g)	ODC (mg GAE/g)	FC (mg CAT/g)
78.55 ± 1.67	80.83 ± 2.69	66.77 ± 1.96

The results are presented as mean ± standard deviation (n = 3).

**Table 3:** Evaluation of antioxidant activities of the GS blend extract by distinct colorimetric chemical assays.

ABTS (mmol TE/g)	DPPH (mmol TE/g)	FRAP (mmol TE/g)
1.035 ± 0.04	0.574 ± 0.03	0.689 ± 0.02

DPPH, DPPH^●^ scavenging capacity; ABTS, ABTS^●+^ scavenging capacity; FRAP, Ferric-reducing antioxidant power; TE, Trolox equivalents. Data are presented as mean ± SD (n = 3).

**Table 4:** Evaluation of anti-aging and depigmenting activities determined by colorimetric enzymatic assays.

% Inhibition Elastase	% Inhibition Tyrosinase
37.78 ± 2.26	45.94 ± 0.63

The results are presented as mean ± standard deviation (n = 3) and are expressed in % inhibition.

**Table 5:** Pearson correlation. (TPC: total phenolic content; ODC: *ortho*-diphenol content; FC: flavonoid content; FRAP assay; DPPH assay; ABTS assay; TYR: anti-tyrosinase assay; ELAS: anti-elastase assay).

	TPC	ODC	FC	FRAP	DPPH	ABTS	TYR	ELAS
**TPC**	1.00	0.89	0.86	0.54	−0.37	0.96	0.24	−0.96
**ODC**	0.89	1.00	1.00	0.09	−0.76	0.98	0.66	−0.72
**FC**	0.86	1.00	1.00	0.02	−0.80	0.96	0.71	−0.68

## Data Availability

Data is contained within the article.
